# Changes in Skin Flavanol Composition as a Response to Ozone-Induced Stress during Postharvest Dehydration of Red Wine Grapes with Different Phenolic Profiles

**DOI:** 10.1021/acs.jafc.0c04081

**Published:** 2020-09-25

**Authors:** Susana Río Segade, Ana Belén Bautista-Ortín, Maria Alessandra Paissoni, Simone Giacosa, Vincenzo Gerbi, Luca Rolle, Encarna Gómez-Plaza

**Affiliations:** ‡ Dipartimento di Scienze Agrarie, Forestali e Alimentari, Università di Torino, Largo Paolo Braccini 2, 10095 Grugliasco, Turin, Italy; § Department of Food Science and Technology, Faculty of Veterinary, University of Murcia, 30071 Murcia, Spain

**Keywords:** ozone exposure, dehydration process, flavanols, postharvest treatments, red wine grapes

## Abstract

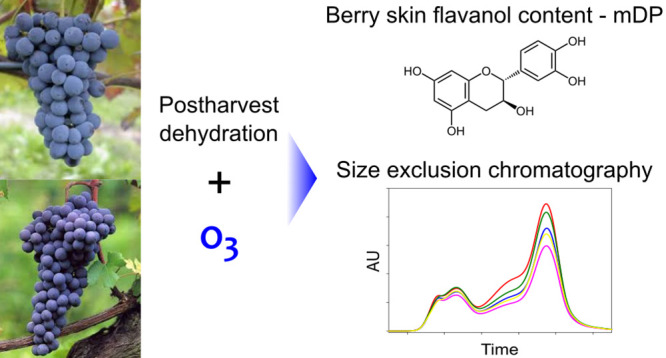

In this study, the combined effect of partial postharvest dehydration and long-term ozone treatment was evaluated at 10 and 20% weight loss as a strategy to induce compositional changes in grape skin flavanols. Two separate trials were carried out in thermohygrometric-controlled chambers at 20 °C and 70% relative humidity. The first trial was conducted under an ozone-enriched atmosphere at 30 μL/L, whereas the second trial was performed under an air atmosphere as a control. Two red wine grape varieties were studied, Barbera and Nebbiolo (*Vitis vinifera* L.), for their different phenolic composition. Berry skin flavanol composition was determined by high-performance liquid chromatography after phloroglucinolysis and size-exclusion chromatography. The results showed that dehydration and ozone effects were variety-dependent. In Barbera skins, being characterized by lower proanthocyanidin contents, the two effects were significant and their combination showed interesting advantages related to lower proanthocyanidin loss as well as higher prodelphinidin and lower galloylation percentages. In Nebbiolo, skin flavanol composition was barely affected.

## Introduction

The composition and content of flavan-3-ols in red wines are gaining increasing interest in the last few years as a consequence of their direct impact on important sensory properties, such as bitterness, astringency, and structure.^1^ In addition, they play a key role in color stability during winemaking and wine aging.^2^ Some studies have highlighted that both flavanol composition and content are related to the quality and market value of wines.^3,4^ Particularly, premium wines have a higher content of flavanols, with a profile mainly composed of highly polymerized compounds. Taking into account that grape skin proanthocyanidins own a high mean degree of polymerization of up to 50 units,^5^ postharvest strategies and oenological techniques enhancing the accumulation and extraction of grape skin proanthocyanidins, respectively, are of great relevance in the wine industry to increase the wine quality grade allocation.

In berry skins, flavanols are accumulated until véraison and then progressively decrease during ripening. The competition of flavanol and anthocyanin biosynthetic pathways for intermediate metabolites (cyanidin and delphinidin) and oxidation phenomena may contribute to this decrease.^6^ Nevertheless, the evolution of each flavanol fraction (monomeric, oligomeric, and polymeric) in the skins is variety-dependent.^6,7^ When extended ripening occurs, skin proanthocyanidins increase again as a metabolic response to osmotic stress.^8^ The partial water loss of grape berries can also be conducted after harvest either under uncontrolled environmental conditions (off-vine sun exposure or in closed naturally ventilated facilities) or in thermohygrometric-controlled chambers.^9^ Among postharvest strategies, grape dehydration is a dynamic physical process that can be used to produce dry wines with special features. During postharvest grape dehydration, besides the concentration effect on primary and secondary metabolites, grape berries are still metabolically reactive to water stress. Particularly, monomeric and oligomeric flavanols show a decreasing trend probably as a result of an increased activity of oxidative enzymes without renewal through synthesis. In fact, leucoanthocyanidin reductase is involved in the flavanol biosynthetic pathway, and no expression changes were observed during the dehydration process.^10^ Nevertheless, metabolic responses are strongly influenced by genotype, dehydration conditions, and stress intensity.^11−13^

In the wine industry, the development of toxin-producing molds is a major problem concerning the wines obtained from dehydrated grapes. Sulfur derivatives are commonly used to prevent the growth of spoilage microorganisms on grape berries. These compounds initially promote water loss by absorption but, once saturated, hinder dehydration.^9^ Other disadvantages associated with the use of sulfur compounds are the occurrence of bleaching injuries in the berry skin, stuck fermentation, and sulfite residues in the wine that can negatively affect human health.

Ozone is a novel and safe alternative because, taking advantage of its strong oxidant activity, it is used for sanitizing purposes without leaving chemical residues on the grape berry surface.^14−16^ Another particularly interesting aspect is that, during postharvest partial grape dehydration, ozone exposure activates antioxidant enzymes at the same time that it inhibits the oxidant activity of polyphenoloxidase and lipoxygenase.^17^ Therefore, ozone could play a protective role against the loss of flavanols by oxidation.

After harvest, ozone exposure can induce important changes in the still active secondary metabolism of grapes, leading to an enhanced synthesis and accumulation of phenolic compounds, such as anthocyanins and stilbenes.^18,19^ This ozone-induced metabolic response is a chemical defense mechanism against the abiotic oxidative stress, which consists of the activation of biosynthetic pathways encouraging the accumulation of low-molecular-weight antioxidant compounds. With regard to flavanols, changes have been reported in short-term postharvest ozone-exposed fresh grapes, showing significantly increased catechin contents but slightly decreased epicatechin.^20^ Moreover, the postharvest ozone exposure of grape berries can promote skin cell wall degradation, facilitating the extractability of oligomeric flavanols and proanthocyanidins.^18^

The combined effect of oxidative and water stresses, induced by wine grape exposure to gaseous ozone during long-term postharvest dehydration, on the content and composition of flavanols in berry skins was scarcely studied. Only one work has been published highlighting that the ozone effect on both the total content, evaluated by spectrophotometric methods, and extraction yield of oligomeric flavanols and proanthocyanidins is variety-dependent.^21^

With the aim of better knowing and understanding these compositional changes of skin flavanols, in the present study, two different and specific analytical approaches were used: the phloroglucinolysis method and size-exclusion chromatography (SEC). Furthermore, the possible influence of the variety–environment interaction was studied using the same environmental conditions of dehydration under an ozone-enriched atmosphere for two wine grape varieties characterized by different phenolic profiles.

## Materials and Methods

### Grape Samples and Dehydration Process

Nebbiolo and Barbera red wine grapes (*Vitis vinifera* L.) from the same vineyard, located in the Piedmont region (Cuneo province, northwest Italy) and cultivated under similar management practices, were harvested at technological maturity (about 24 °Brix) in 2015. For each grape variety, a set of fresh grape berries (about 5 kg) were randomly selected (fresh sample). Then, whole bunches were divided in small clusters of 5–6 berries each and randomly arranged in a single layer into 12 small perforated boxes (20 × 30 cm, about 1.5 kg of clusters each), for correct aeration, in which they were partially dehydrated as follows. Six sample boxes were dehydrated under an ozone-enriched atmosphere at 30 μL/L using a continuous 120 m^3^/h flow produced by an ozone generator (C32-AG, Industrie De Nora Spa, Milan, Italy) with a nominal production capacity of 32 g of O_3_/h:^18,21^ the first three boxes up to 10% weight loss (WL) and the other three boxes up to 20% WL. At the same time, the other six sample boxes were dehydrated under an air atmosphere up to the same WL (10 and 20%) and were used as a control. The dehydration process was conducted into thermohygrometrically controlled chambers at 20 ± 2 °C temperature and 70 ± 5% relative humidity.^21^

### Total Skin Phenolic Compound Extraction and Determination

The determination of total skin phenolic compounds in fresh grape berries was conducted following the extraction procedure reported by Río Segade et al.^21^ For each wine grape variety, the berry skins from 10 randomly selected berries were separated from the mesocarp, weighed, and quickly immersed into 50 mL of a hydroalcoholic buffer solution at pH 3.2 consisting of 5 g/L tartaric acid, 12% (v/v) ethanol, and 2 g/L sodium metabisulfite. To complete the extraction of phenolic compounds from skins, the suspensions were homogenized at 8000 rpm for 1 min using an Ultraturrax T25 high-speed homogenizer (IKA Labortechnik, Staufen, Germany) and then centrifuged for 15 min at 3000*g* at 20 °C in a PK 131 centrifuge (ALC International, Milan, Italy). The extraction was performed by triplicate.

In the skin extracts obtained, total flavonoids (TF), total nonanthocyanin flavonoids (FNA), total anthocyanins (TA), proanthocyanidins after acid hydrolysis (PRO), and flavanols reactive to vanillin (FRV) were determined spectrophotometrically^21^ using an UV-1800 spectrophotometer (Shimadzu Corporation, Kyoto, Japan). To characterize the phenolic composition of fresh berry skins, the results were expressed as milligrams per gram of skin (wet weight) using standards of (+)-catechin (Sigma-Aldrich, St. Louis, MO, U.S.A.) for TF, FNA, and FRV, malvidin-3-glucoside chloride (Extrasynthèse, Genay, France) for TA, and cyanidin chloride (Sigma-Aldrich) for PRO.

### Skin Flavanol Extraction

The extraction was performed following the method described by Busse-Valverde et al.^22^ For each grape variety and sample, the mesocarp-free skins were freeze-dried and powdered to evaluate mainly the effects imputable to grape berry exposure to gaseous ozone during withering, thus preventing the possible masking of these effects as a consequence of the different dehydration levels. Three replicates of each freeze-dried and powdered sample (0.8 g) were treated with 10 mL of a 2:1 acetone/water solution at room temperature for 24 h at 200 rpm on an orbital shaker. The extraction was carried out in covered vials saturated with nitrogen and in the dark to minimize oxidation. Then, the extract was evaporated to dryness at 35 °C and redissolved in 1 mL of methanol.

### Flavanol Determination Using the Phloroglucinolysis Method

Skin proanthocyanidins (PAs) were determined according to the method proposed by Kennedy and Jones,^23^ with some modifications.^22^ The phloroglucinolysis reagent consisted of a 0.2 M HCl solution in methanol, containing 100 g/L phloroglucinol and 20 g/L ascorbic acid. The methanolic extract was treated with the phloroglucinolysis reagent (1:1) for 20 min at 50 °C in a water bath, and then the reaction was stopped by adding 2 volumes of 0.2 M aqueous sodium acetate. High-performance liquid chromatography (HPLC) analysis was carried out using a Waters 2695 system (Waters, Milford, MA, U.S.A.), equipped with a Waters 2996 photodiode array detector, and following the conditions described by Ducasse et al.^24^ A sample volume of 10 μL was injected on an Atlantis dC18 column (250 × 4.6 mm, 5 μm) coupled to a guard column of the same material (20 × 4.6 mm, 5 μm, Waters). The column temperature was set to 30 °C. The mobile phase consisted of water/formic acid (98:2, v/v) as solvent A and acetonitrile/solvent A (80:20, v/v) as solvent B, working at 0.8 mL/min flow rate. Gradient elution was performed, starting at 0% of B for 5 min, increasing from 0 to 10% of B in 30 min, and increasing from 10 to 20% of B in 30 min, followed by the washing and re-equilibration of the column. The total PA content, the mean degree of polymerization (mDP), and the percentage of each constitutive unit were determined from the absorbance value at 280 nm. (+)-Catechin standard was used for the quantification of PA cleavage products. The total PA content was expressed as milligrams per gram of skin [dry weight (dw)]. The mDP was calculated as the molar ratio of the sum of all of the flavanol units produced by phloroglucinolysis (phloroglucinol adducts and monomers) to the sum of monomeric forms. The percentage of galloylation (G) was calculated as the ratio of the sum of galloylated flavanols to the sum of all flavanols, and the percentage of prodelphinidins (ProDP) was calculated as the ratio of (−)-epigallocatechin content to the sum of all flavanols.

### Flavanol Determination by SEC

The analysis of methanolic skin extracts by SEC was carried out according to the method reported by Kennedy and Taylor,^25^ with some modifications.^26^ Two polystyrene–divinylbenzene PLgel columns (300 × 7.5 mm, 5 μm) of 500 Å (effective molecular mass range of 500–30 000 g/mol using polystyrene standards) and 100 Å (effective molecular mass range of up to 4000 g/mol using polystyrene standards) were connected in series, and the temperature was set to 60 °C. A guard column containing the same material (50 × 7.5 mm, 5 μm) was used. All columns were purchased from Polymer Laboratories (Amherst, MA, U.S.A.). The amount of sample injected corresponded to 40 μg. The mobile phase consisted of *N*,*N*-dimethylformamide containing 1% (v/v) glacial acetic, 5% (v/v) water, and 0.15 M lithium chloride, working in isocratic mode at 1 mL/min flow rate. The determination was performed at 280 nm. The total area corresponding to PAs and the area associated with each PA fraction (high, medium, and low molecular mass) were calculated as well as the ratio of each fraction to total area.

### Statistical Analysis

Data were statistically analyzed using R 3.6.2 software.^27^ Analysis of variance (ANOVA) was carried out to evaluate significant differences (*p* < 0.05) between treatments (ozone and air) at the same dehydration level (10 and 20% WL) and among dehydration levels (fresh grape berries and 10 and 20% WL) for the same treatment on Barbera and Nebbiolo varieties individually. If significant differences (*p* < 0.05) were found, a Tukey honest significant difference (HSD) post hoc test was used. Moreover, two-way ANOVA was used for evaluating the effects of the treatment, dehydration, and their interaction for each variety.

Discriminant analysis (DA) was performed using the SPSS statistics software package (version 25.0, IBM Corporation, Armonk, NY, U.S.A.) to classify samples according to the treatment (fresh grape, air-exposed, and ozone-treated samples; *n* = 15) or to the dehydration level (fresh grape and 10 and 20% WL; *n* = 15), using the flavanol content and composition variables. Each variety was considered individually to avoid bias given by the different compositional features.

## Results and Discussion

### Fresh Berry Skin Phenolic Composition

**Figure 1:**
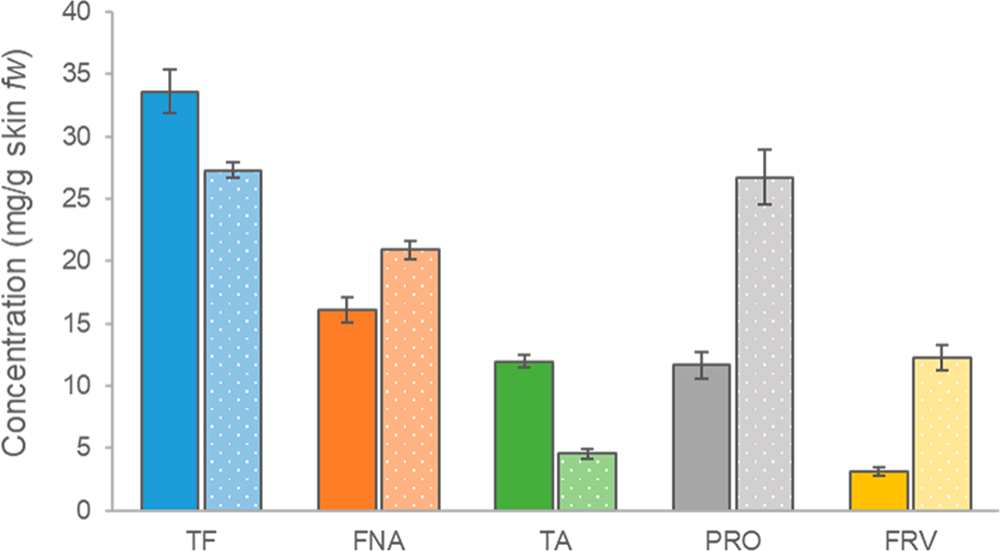
Phenolic composition of Barbera (solid color bars) and Nebbiolo (spotted color bars) fresh grape skins expressed as milligrams per gram of fresh skins. TF, total flavonoids; FNA, total nonanthocyanin flavonoids; TA, total anthocyanins; PRO, proanthocyanidins; FRV, flavanol reactive to vanillin; and fw, fresh weight.

Figure 1 shows the total content of phenolic compounds in fresh berry skins for Barbera and Nebbiolo red wine grapes. Barbera berry skins presented a higher content of total flavonoids with respect to Nebbiolo when considering skin fresh weight (TF, 33.60 versus 27.30 mg/g of berry skins fw, respectively). In addition, a different ratio between flavanols and anthocyanins was found for the two varieties, as already previously reported.^18^ In fact, Barbera skins own a lower quantity of nonanthocyanin flavonoids when compared to Nebbiolo (FNA, 16.07 versus 20.88 mg/g of berry skins fw), because a larger part of flavonoids was represented by total anthocyanins (TA, 11.95 versus 4.54 mg/g of berry skins fw). With regard to flavanols, both polymeric (PRO) and oligomeric (FRV) forms were lower in Barbera with respect to Nebbiolo berry skins (11.66 versus 26.72 and 3.10 versus 12.29 mg/g of berry skins fw for PRO and FRV, respectively, for Barbera and Nebbiolo). Nevertheless, the ratio FRV/PRO was higher in Nebbiolo with respect to Barbera (0.46 versus 0.27, respectively). A low FRV/PRO index may evidence higher molecular complexity related to the spatial conformation and sterical hindrance, i.e., more branched structure, or to the involvement of nucleophilic sites in interflavan linkages to form polymeric structures, hindering the electrophilic addition of vanillin.^6^ The results obtained are in accordance with the different flavanol composition features reported for these.^6,18^ Moreover, the variety differences found in the skin flavanol content and composition, assessed by spectrophotometric assays, have promoted the selection of Barbera and Nebbiolo for assessing the impact of the ozone treatment during partial grape dehydration on varieties with quite different skin flavanol profiles.

### Total Flavanols and Mean Degree of Polymerization during Grape Dehydration

The impact of continuous exposure of Barbera and Nebbiolo grape berries to gaseous ozone on the flavanol content and composition was evaluated during postharvest partial dehydration. Particularly, the ozone effect has been assessed on berries dehydrated at 10 and 20% WL with respect to the dehydration under an air atmosphere as the control. Moreover, it is possible to evidence if flavanols evolve similarly during withering under ozone-enriched and air atmosphere or, instead, ozone has promoted the synthesis, loss, or transformation of these compounds. It is important to evidence that control samples (air-treated) required 5 days more than those ozone-exposed to reach the same dehydration level (7 and 12 days for 10% WL under ozone-enriched and air atmosphere, respectively, and 15 and 20 days for 20% WL under ozone-enriched and air atmosphere, respectively), despite using the same temperature and relative humidity conditions. This may be due to the absence of air flow produced by the ozone generator during the treatment of control samples.

The results obtained for Barbera and Nebbiolo berry skins are shown in Tables 1 and 2, respectively. In fresh grapes, Barbera berry skins (Table 1) were significantly less rich in total flavanols, measured by the phloroglucinolysis assay (phl-PAs, dw), than Nebbiolo skins (Table 2), in agreement with spectrophotometric assays (Figure 1). In our experimental conditions, Nebbiolo showed no significant differences in the phl-PA content with dehydration and ozone treatment. In contrast, phl-PAs decreased in Barbera at 20% WL, and this reduction was more relevant for air condition than for an ozone-enriched atmosphere (−5.92 and −2.55 mg/g of skins dw for 20% WL air and 20% WL ozone, respectively, compared to fresh grapes; *p* < 0.001). Therefore, ozone reduced the loss in total phl-PA content from −46 to −20%. In a previously published study,^21^ no differences were reported at 10% WL for oligomeric and polymeric flavanols (estimated as FRV and PRO indices, respectively) for both the varieties and at 20% WL for Nebbiolo using the same conditions as the present study for dehydration and ozone treatment. In contrast, a strong decrease was found particularly for PRO index in Barbera at 20% WL, but it was more evident for ozone-treated grapes, apparently in disagreement with the results of the present study.

**Table 1:** Proanthocyanidin (PA) Composition of Barbera Grape Skins before Dehydration and after 10 and 20% Weight Loss under Air and Ozone-Enriched Atmosphere According to Phloroglucinolysis Analysis^a^

weight loss	0	10			20			sign^b^		sign^c^		
treatment	fresh grapes	air	ozone	sign^a^	air	ozone	sign^a^	air	ozone	treatment	dehydration	interaction
phl-PAs (mg/g of skin dw)	12.81 ± 0.36 aA	12.33 ± 0.38 a	11.61 ± 0.19 B	∗	6.89 ± 0.76 b	10.26 ± 0.52 C	∗∗	∗∗∗	∗∗∗	∗∗∗	∗∗∗	∗∗∗
mDP	9.99 ± 0.08 bC	10.46 ± 0.28 b	12.61 ± 0.48 A	∗	15.74 ± 0.46 a	11.76 ± 0.18 B	∗∗∗	∗∗∗	∗∗∗	∗∗∗	∗∗∗	∗∗∗
G (%)	2.77 ± 0.47 bA	2.71 ± 0.06 b	2.37 ± 0.12 B	∗	3.33 ± 0.22 a	2.35 ± 0.06 B	∗∗	∗∗	∗∗	∗∗∗	∗∗	∗∗∗
ECG (μM)	96.65 ± 5.15 aA	89.80 ± 4.74 a	74.18 ± 2.58 B	∗∗	61.06 ± 3.52 b	65.02 ± 1.63 C	ns	∗∗∗	∗∗∗	∗∗∗	∗∗∗	∗∗
ProDP (%)	17.18 ± 0.13 bB	19.97 ± 0.13 a	16.71 ± 0.99 B	∗	17.69 ± 0.46 b	21.74 ± 1.29 A	∗∗	∗∗	∗∗∗	∗∗	ns	∗∗∗
EGC (μM)	593 ± 21 a	662 ± 8 a	554 ± 5	∗∗∗	309 ± 51 b	602 ± 66	∗∗	∗∗∗	ns	∗∗	ns	∗∗∗
Terminal Units												
C_t_ (%)	71.35 ± 0.47 bB	73.47 ± 1.01 b	82.05 ± 2.10 A	∗∗	83.35 ± 3.10 a	84.78 ± 1.25 A	ns	∗∗∗	∗∗∗	∗∗∗	∗∗∗	∗∗
EC_t_ (%)	28.65 ± 0.47 aA	26.53 ± 1.01 a	17.95 ± 2.10 B	∗∗	16.65 ± 3.10 b	15.22 ± 1.25 B	∗	∗∗∗	∗∗∗	∗∗∗	∗∗∗	∗∗
Extension Units												
C_ext_ (%)	1.26 ± 0.17	1.10 ± 0.08	1.24 ± 0.11	ns	1.06 ± 0.20	1.48 ± 0.02	∗∗	ns	ns	∗	ns	ns
EC_ext_ (%)	76.57 ± 0.41 aA	73.83 ± 0.88 b	76.98 ± 0.44 A	∗∗	77.54 ± 0.81 a	72.18 ± 1.33 B	∗∗	∗∗	∗∗∗	∗	ns	ns
EGC_ext_ (%)	19.09 ± 0.16 bB	22.08 ± 1.01 a	19.21 ± 0.43 B	∗	17.84 ± 1.03 b	23.76 ± 1.38 A	∗∗	∗∗	∗∗∗	∗∗	ns	∗∗∗
ECG_ext_ (%)	3.08 ± 0.00 bA	2.90 ± 0.06 b	2.57 ± 0.12 B	∗∗	3.56 ± 0.23 a	2.57 ± 0.07 B	∗∗	∗∗	∗∗∗	∗∗∗	∗∗	∗∗

a All data are expressed as the average value ± standard deviation (*n* = 3). Sign: ∗, ∗∗, ∗∗∗, and ns indicate significance at *p* < 0.05, 0.01, 0.001, and not a significant difference, respectively, between the treatments (ozone treatment or air condition, sign^a^) at each dehydration level and between the dehydration levels (fresh grapes and 10 and 20% weight loss, sign^b^) for air condition or ozone treatment according to one-way ANOVA. Different lowercase letters within the same row indicate significant differences (sign^b^) among fresh grape and 10 and 20% WL for air-exposed samples according to the Tukey HSD test (*p* < 0.05). Different capital letters within the same row indicate significant differences (sign^b^) among fresh grape and 10 and 20% WL for ozone-treated samples according to the Tukey HSD test (*p* < 0.05). Sign^c^ indicates significance according to two-way ANOVA with treatment and dehydration as factors and their interaction. Abbreviation: phl-PAs, total proanthocyanidins estimated by the phloroglucinolysis method; mDP, mean degree of polymerization; G, galloylation; ECG, epicatechin gallate; ProDP, prodelphinidins; EGC, epigallocatechin; C_t_, catechin terminal unit; EC_t_, epicatechin terminal unit; C_ext_, catechin extension unit; EC_ext_, epicatechin extension unit; EGC_ext_, epigallocatechin extension unit; ECG_ext_, epicatechin gallate extension unit; and dw, dry weight.

**Table 2:** Proanthocyanidin (PA) Composition of Nebbiolo Grape Skins before Dehydration and after 10 and 20% Weight Loss under Air and Ozone-Enriched Atmosphere According to Phloroglucinolysis Analysis^a^

weight loss	0	10			20			sign^b^		sign^c^		
treatment	fresh grapes	air	ozone	sign^a^	air	ozone	sign^a^	air	ozone	treatment	dehydration	interaction
phl-PAs (mg/g of skin dw)	29.29 ± 1.31	26.62 ± 2.23	26.99 ± 0.23	ns	27.18 ± 1.44	27.63 ± 1.26	ns	ns	ns	ns	ns	ns
mDP	16.55 ± 0.04 b	18.69 ± 0.67 a	16.96 ± 0.38	∗	17.02 ± 0.26 b	17.72 ± 0.90	ns	∗∗	ns	∗	ns	∗∗
G (%)	0.72 ± 0.04 bC	0.93 ± 0.07 a	0.92 ± 0.04 B	ns	1.00 ± 0.04 a	1.12 ± 0.06 A	∗	∗∗	∗∗∗	∗∗∗	∗∗∗	∗
ECG (μM)	56.62 ± 1.34 bC	65.96 ± 0.90 ab	66.27 ± 2.19 B	ns	72.92 ± 6.94 a	82.41 ± 2.05 A	ns	∗∗	∗∗∗	∗∗∗	∗∗∗	ns
ProDP (%)	47.48 ± 1.45	47.11 ± 0.15	47.11 ± 0.15	ns	47.15 ± 0.43	46.18 ± 0.96	ns	ns	ns	ns	ns	ns
EGC (μM)	3722 ± 56 A	3357 ± 293	3407 ± 60 B	ns	3341 ± 127	3412 ± 135 B	ns	ns	∗∗	∗	ns	ns
Terminal Units												
C_t_ (%)	63.66 ± 1.34 bB	68.91 ± 0.49 a	66.50 ± 0.02 A	∗∗	64.32 ± 0.45 b	67.10 ± 1.03 A	∗	∗∗∗	∗	∗∗∗	∗∗	∗∗∗
EC_t_ (%)	36.34 ± 1.34 aA	31.09 ± 0.49 b	33.50 ± 0.02 B	∗∗	35.68 ± 0.45 a	32.90 ± 1.03 B	∗	∗∗∗	∗	∗∗∗	∗∗	∗∗∗
Extension Units												
C_ext_ (%)	2.61 ± 0.14 bB	3.04 ± 0.02 a	2.91 ± 0.00 A	∗∗∗	2.94 ± 0.23 ab	2.43 ± 0.02 B	∗	∗	∗∗∗	∗∗	∗∗	∗
EC_ext_ (%)	46.08 ± 1.82	46.21 ± 0.21	46.00 ± 0.34	ns	47.18 ± 0.98	47.43 ± 0.99	∗∗	ns	ns	ns	ns	ns
EGC_ext_ (%)	50.54 ± 1.64	49.77 ± 0.26	50.11 ± 0.39	ns	48.81 ± 0.79	48.95 ± 0.94	ns	ns	ns	ns	ns	ns
ECG_ext_ (%)	0.77 ± 0.05 bC	0.98 ± 0.07 a	0.98 ± 0.04 B	ns	1.06 ± 0.04 a	1.18 ± 0.06 A	∗	∗∗	∗∗∗	∗∗∗	∗∗∗	ns

a All data are expressed as the average value ± standard deviation (*n* = 3). Sign: ∗, ∗∗, ∗∗∗, and ns indicate significance at *p* < 0.05, 0.01, 0.001, and not a significant difference, respectively, between the treatments (ozone treatment or air condition, sign^a^) at each dehydration level and between the dehydration levels (fresh grapes and 10 and 20% weight loss, sign^b^) for air condition or ozone treatment according to one-way ANOVA. Different lowercase letters within the same row indicate significant differences (sign^b^) among fresh grape and 10 and 20% WL for air-exposed samples according to the Tukey HSD test (*p* < 0.05). Different capital letters within the same row indicate significant differences (sign^b^) among fresh grape and 10 and 20% WL for ozone-treated samples according to the Tukey HSD test (*p* < 0.05). Sign^c^ indicates significance according to two-way ANOVA with treatment and dehydration as factors and their interaction. Abbreviation: phl-PAs, total proanthocyanidins estimated by the phloroglucinolysis method; mDP, mean degree of polymerization; G, galloylation; ECG, epicatechin gallate; ProDP, prodelphinidins; EGC, epigallocatechin; C_t_, catechin terminal unit; EC_t_, epicatechin terminal unit; C_ext_, catechin extension unit; EC_ext_, epicatechin extension unit; EGC_ext_, epigallocatechin extension unit; ECG_ext_, epicatechin gallate extension unit; and dw, dry weight.

The content of phenolic compounds, when expressed per wet weight, increases (PAs included) with postharvest grape dehydration mainly as a result of the concentration effect.^28^ In contrast, if the results expressed per dry weight (dw) or per berry basis are considered, variable results have been found depending upon the variety, dehydration level, and environmental conditions.^10,11,21,29^ The decrease of PAs is consistent with their oxidation.^30,31^ The flavanol biosynthetic pathway has been demonstrated to not be induced by postharvest dehydration,^10^ and the degradation of monomeric and oligomeric flavanols by both chemical and enzymatic oxidations may lead to a decrease of flavanols without any replacement by neo-synthesis.^10,11^ Polyphenoloxidase (PPO)-encoding genes were proven to be upregulated during dehydration of Corvina and Raboso Piave grapes,^10,32^ and an increased PPO activity was reported for the Aleatico variety.^11^ Nevertheless, the oxidative stress promoted by ozone could activate different antioxidant enzymes, such as superoxide dismutase, catalase, ascorbate peroxidase, and guaiacol peroxidase, inhibiting at the same time the polyphenoloxidase activity.^17^

In fresh grapes, PAs of Nebbiolo skins presented a higher mDP value than those of Barbera and different trends were highlighted for the two varieties during postharvest dehydration. According to the two-way ANOVA, mDP was influenced by both treatment and dehydration in Barbera berry skins, whereas only the treatment effect was significant in Nebbiolo (Tables 1 and 2). Generally, the mDP value increased when dehydration progressed, but it did significantly only for Barbera. Furthermore, the treatment and its interaction with dehydration influenced mDP differently for both of the varieties (treatment, *p* < 0.05 and *p* < 0.001; interaction, *p* < 0.01 and *p* < 0.001, for Nebbiolo and Barbera, respectively). In fact, the mDP value increased in Barbera ozone-treated grapes at both WLs (+2.61 and +1.76 for 10 and 20% WL, respectively, compared to fresh grapes; *p* < 0.001; Table 1). When dehydration was conducted under an air atmosphere, no change was reported at 10% WL for mDP, whereas the increase was remarkable at 20% WL (+5.75 with respect to fresh grapes; *p* < 0.001). In contrast, the mDP value of Nebbiolo skins was increased by dehydration only in grape berries dehydrated at 10% WL under air exposure with respect to both fresh grapes (+2.14; *p* < 0.01) and the corresponding ozone-treated samples (+1.67; *p* < 0.05; Table 2).

Oligomeric and polymeric PA hydrolysis has been suggested during postharvest grape dehydration.^10^ As a consequence of PA degradation, a decrease of the mDP value in skin PAs was expected.^28−30^ Although the results of the present study seem to be in contrast with what was previously reported on the mDP value during grape dehydration, PAs may react to different extents, increasing the linear chain structure, usually by intermolecular bonding with the terminal unit.^33,34^ On one hand, a higher mDP value could be expected by the formation of longer linear chains, even though intramolecular reactions can occur without mDP change, and on the other hand, intermolecular reactions may also happen with other flavanol extension units, giving a branched structure that is no longer sensible to acid cleavage or accessible for the reaction. Therefore, these last mentioned reactions lead to a mDP underestimation.^31,34^ In a wine-like medium, Lee^31^ showed that the oxidation mechanism may lead to dynamic changes in the chain length, influencing mDP. This change is affected by the PA composition, the presence of monomeric flavanols, and the increase of unidentified compounds.^30,31^ Although no data are available on the effect of ozone on the mDP value, the different variations observed with respect to air exposure, at both 10 and 20% WL for the same variety and for Barbera and Nebbiolo varieties at the same dehydration level, seem to confirm these dynamic changes and a variety dependence related to flavanol composition (Tables 1 and 2).

### SEC

To better understand the results obtained, SEC was performed.^25,26^ In fact, SEC allows for better evaluation of the effect of grape dehydration on PAs, given the lower effectiveness of the phloroglucinolysis method in determining oxidized PAs.^25,34^ Using SEC, the advantages are both to include constituents that were not converted to their monomeric subunits and to provide information on the mass distribution of the different PAs.

**Figure 2:**
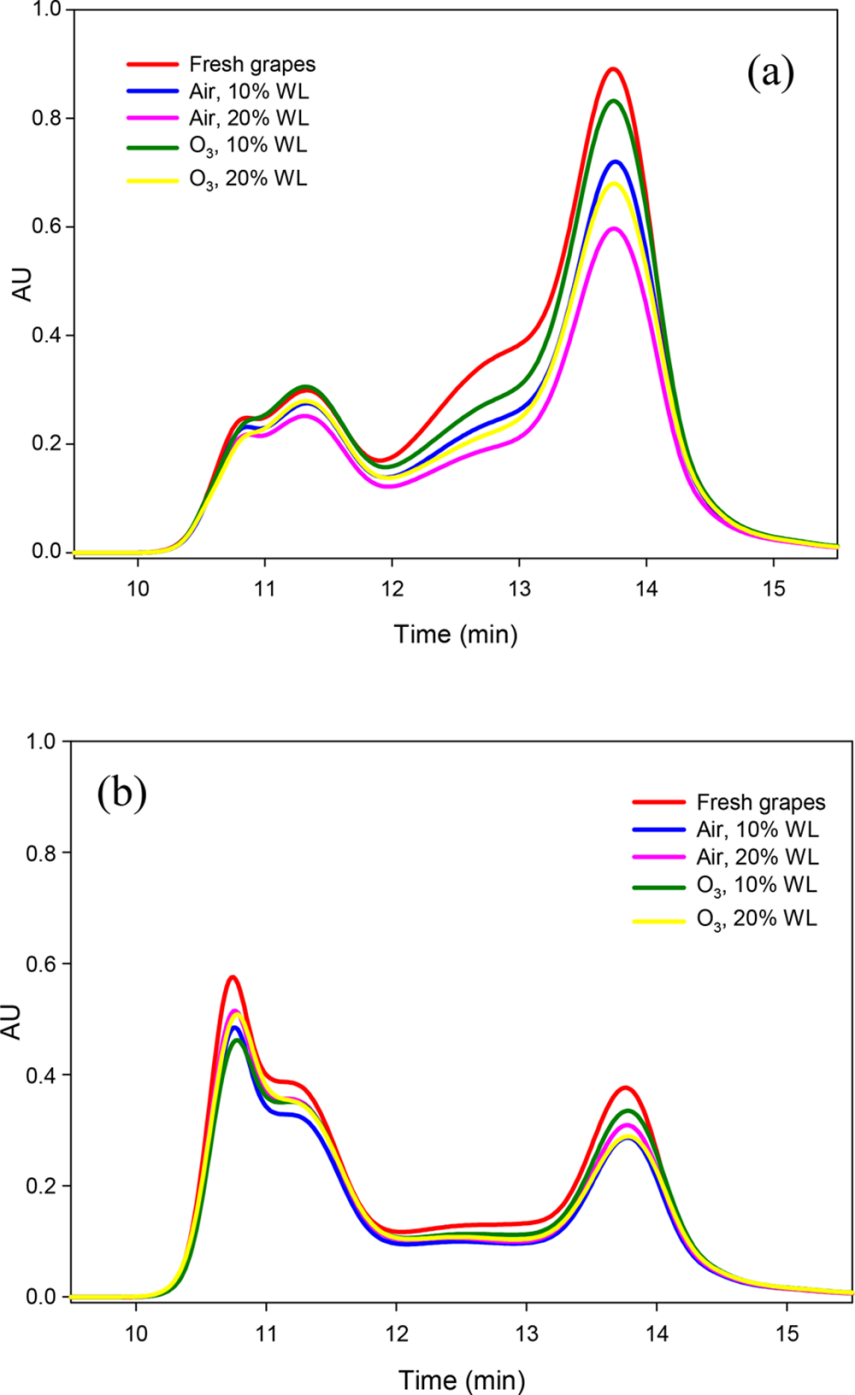
SEC profile of (a) Barbera and (b) Nebbiolo grapes before (fresh samples) and after dehydration at 10 and 20% weight loss under air and ozone-enriched atmosphere.

SEC chromatograms are reported in Figure 2, and total area corresponding to PAs and the area associated with each molecular mass fraction are shown in Table 3. Fraction 1 (F1) corresponds to high-molecular-mass PAs (high-m, range of 840 000–30 000 g/mol, eluting from 10 to 11.8 min); fraction 2 (F2) corresponds to medium-molecular-mass PAs (medium-m, range of 30 000–1000 g/mol, eluting from 11.8 to 13.6 min); and fraction 3 (F3) corresponds to low-molecular-mass PAs (low-m, <1000 g/mol, including flavanol trimers, dimers, monomers, and anthocyanins, eluting from 13.6 to 16 min).

**Table 3:** Total Area Determined by SEC for Proanthocyanidins (PAs) and Area Corresponding to High-Molecular-Mass PAs, Medium-Molecular-Mass PAs, and Low-Molecular-Mass PAs for Barbera and Nebbiolo Grape Skins before Dehydration and after 10 and 20% Weight Loss under Air and Ozone-Enriched Atmosphere^a^

weight loss	0	10		20	
treatment	fresh grapes	air	ozone	air	ozone
Barbera					
total area	1.458	1.183	1.353	1.016	1.145
area high-m PAs	0.108 (7.43)	0.099 (8.37)	0.104 (7.66)	0.096 (9.40)	0.093 (8.14)
area medium-m PAs	0.573 (39.27)	0.449 (37.94)	0.510 (37.71)	0.388 (38.16)	0.437 (38.15)
area low-m PAs	0.777 (53.29)	0.635 (53.68)	0.739 (54.63)	0.533 (52.45)	0.615 (53.71)
Nebbiolo					
total area	0.965	0.787	0.860	0.848	0.849
area high-m PAs	0.252 (26.14)	0.211 (26.79)	0.204 (23.77)	0.227 (26.78)	0.228 (26.88)
area medium-m PAs	0.405 (41.93)	0.334 (42.45)	0.369 (42.93)	0.360 (42.49)	0.364 (42.92)
area low-m PAs	0.308 (31.92)	0.242 (30.76)	0.286 (33.30)	0.261 (30.74)	0.256 (30.20)

a The numbers in parentheses indicate the ratio of each PA fraction to total area: high-m, high molecular mass; medium-m, medium molecular mass; and low-m, low molecular mass.

In the two varieties studied, a decrease in total area during dehydration was found (from −7.2 to −30.3% with respect to fresh grapes, for both varieties). In Barbera skins, a higher reduction of the total area was reported for air-exposed samples with respect to ozone-treated samples (−30.3 and −21.5% for air and ozone, respectively, at 20% WL). Anyway, the profile and trend of the studied varieties were different as a result of the different contents and compositions of PAs. In Nebbiolo skins, a slight decrease was observed for all fractions with respect to fresh grapes, independent of the molecular mass. Nevertheless, some differences were observed. With regard to high-m PAs (F1), a decrease in terms of area was evident at 10% WL (−16.4 and −19.0% for air and ozone, respectively), whereas a lesser decrease of high-m PAs at 20 WL% may suggest oxidative coupling of PAs during dehydration (−10.0 and −9.6% for air and ozone, respectively). In Barbera, a decrease in area was generally found for F1 during dehydration (from −4.3 to −14.0%). Nevertheless, high-m PAs increased in terms of the ratio with respect to fresh grapes (from +0.2 to +2.0%), counterparted by a decrease of medium-m PAs (from −1.1 to −1.6%, F2). The area corresponding to F2 was reduced in Nebbiolo (from −8.8 to −17.5%) but increased in terms of the ratio with respect to fresh grapes (from +0.5 to +1.0%). With regard to the low-m PA fraction (F3), it was reduced in terms of the area in all samples for both of the varieties (from −15.4 to −31.4%, with respect to fresh grapes), except for grapes dehydrated at 10% WL under an ozone-enriched atmosphere, which showed an increase in terms of the ratio as well (+1.3 and +1.4%, for Barbera and Nebbiolo, respectively).

Ozone-induced changes are related to its strong oxidation potential.^35^ As a consequence, PAs undergo both oxidation by ozone intermediate radicals (such as ^•^O_2_–, HO_2_^•^, ^•^OH, and ^•^O_3_−) and direct reaction based on the Criegee mechanism of ozonolysis.^36^ Most PA ozonolysis products belong to flavonoids (which may explain the increase in total flavonoids and, in our case, low-m PAs), followed by the formation of organic acids that will be finally decomposed in H_2_O and CO_2_.^35,37^

The results obtained by SEC (Figure 2 and Table 3) are quite in agreement with those reported by phloroglucinolysis (Tables 1 and 2). In Barbera, the decrease observed in total area by SEC, when compared to fresh grapes, corresponded mainly to a large reduction in the low-m (F3) and medium-m (F2) fractions as well as to a very small reduction in the high-m fraction (F1), especially in grape berries dehydrated at 20% WL under air exposure, which agree with the lowest PA content and highest mDP value by the phloroglucinolysis method. In Nebbiolo, the greatest differences reported by SEC corresponded to ozone-treated samples at 10% WL with respect to fresh grapes. In this case, the lower F1 and higher F3 fractions in terms of the ratio did not fit well with the similar mDP value observed. In addition, Nebbiolo skins showed a higher decrease in total area for SEC than in the PA content by phloroglucinolysis (from −10.9 to −18.5% and from −5.7 to −9.1% compared to fresh grape, estimated as the SEC total area and phl-PAs, respectively).

### Proanthocyanidin Composition

A compositional study of terminal and extension units was also carried out (Tables 1 and 2 for Barbera and Nebbiolo, respectively). Changes in the terminal units of PAs were found for the two varieties with dehydration under ozone-enriched and air atmosphere. An increase of catechin (C_t_), which is the predominant terminal unit, was reported with respect to fresh grapes, whereas epicatechin (EC_t_) was significantly reduced as a result of the dehydration effect (according to two-way ANOVA, for both *p* < 0.001 and *p* < 0.01 for Barbera and Nebbiolo, respectively). During dehydration, a general increase of catechin units was reported for the Pinot noir variety^29^ and C_t_ for Garnacha tintorera.^28^ Concerning our findings, this increasing trend was confirmed for C_t_ at both 10 and 20% WL in ozone-treated Barbera grapes (+10.70 and +13.43% for 10 and 20% WL, respectively; *p* < 0.001), whereas in air-exposed samples, a significant increase was reported only at 20% WL (+12.00%; *p* < 0.001) with respect to fresh grapes. In Nebbiolo skins, a significantly higher C_t_ percentage was found for both dehydration levels in ozone-treated samples (+2.85 and +3.44% for 10 and 20% WL, respectively; *p* < 0.05) and at 10% WL under air exposure (+5.25%; *p* < 0.001) with respect to fresh grapes. In a wine-like solution, increased C_t_ was reported by Vidal et al.^30^ subsequent to the incorporation of monomers in PAs.

Noteworthy, computation of terminal units also considers monomeric epicatechin and catechin, which may be strongly influenced by dehydration and ozone treatment. In fact, some authors reported a decrease of epicatechin in Merlot and Cabernet sauvignon grapes dehydrated up to 30% WL^38^ and an increase of catechin in Aleatico, Merlot, and Cabernet sauvignon varieties during dehydration.^11,38^ With regard to the present work, the dehydration effect was less remarkable in Nebbiolo, which could be due to a different variety responses, as previously suggested. The flavanol monomer content is influenced by the variety, dehydration level, and environmental conditions.^11^ In fact, both epicatechin and catechin contents decreased after dehydration in Cesanese and Raboso Piave varieties.^10,39^ For ozone treatment, information about the trend of PA compositional units is reported here for the first time. Anyway, the increase of monomeric catechin and the decrease of epicatechin were consistent with those of postharvest ozone shock treatment (12 h) in Grechetto fresh wine grapes.^20^

In Nebbiolo skins, also catechin extension units (C_ext_) were influenced by the ozone treatment and dehydration (according to two-way ANOVA; *p* < 0.01; Table 2), and they particularly increased at 10% WL (+0.43 and +0.30% for air and ozone-treated samples, respectively, compared to fresh grapes). Air-exposed samples showed a significantly higher percentage of C_ext_ with respect to their corresponding ozone-treated samples (+0.13%; *p* < 0.001 and +0.51%; *p* < 0.05, at 10 and 20% WL, respectively). In contrast, in Barbera (Table 1), only ozone-treated grapes at 20% WL reported significantly higher percentages of C_ext_ with respect to air-exposed grapes at 20% WL (+0.42%; *p* < 0.01). In Barbera skins, several changes were also found for epicatechin (EC_ext_) and epigallocatechin (EGC_ext_), although the effect of the treatment varied depending upon the dehydration level. Ozone-treated samples showed significantly higher percentages of EC_ext_ at 10% WL (+3.15%; *p* < 0.01) but lower percentages of EC_ext_ at 20% WL (−5.36%; *p* < 0.01), with respect to air-exposed samples, whereas the opposite happened for EGC_ext_ (−2.87%; *p* < 0.05 and +5.92%; *p* < 0.01, for 10 and 20% WL, respectively; Table 1), confirming the dynamic changes of PAs.^31^ The content of EGC was higher in Nebbiolo than in Barbera, as both total EGC (μM) and the percentage on the total units (ProDP %). Interestingly, these two parameters were not affected by either the treatment or the dehydration in the Nebbiolo variety (*p* > 0.05). Nevertheless, in Barbera skins, the content of EGC was significantly reduced at 20% WL in air-exposed samples (−47.94%; *p* < 0.001), whereas the ozone treatment avoided this decrease (*p* > 0.05). This parameter contributes to smooth perceived astringency in wines because it is negatively related to astringency.^40^

Galloylated PAs are rarely found in grape skins, and their contribution on total constituents of grape skins is low, with a range of 0.72–3.33% in the varieties studied here (Tables 1 and 2), with a lower percentage of galloylation for PAs being reported in Nebbiolo skins than in Barbera. In wines, galloylated PAs are mainly derived from grape seeds and are particularly relevant in terms of astringency sensation.^40^ Nevertheless, these values are in accordance with those found in other red wine grape varieties as recently reviewed by Rousserie et al.^5^

Skin PA galloylation was strongly influenced by both dehydration and treatment in terms of either galloylation percentage (G) or the content of epicatechin gallate (ECG, μM). Taking into account that ECG was not reported as terminal units, these differences are mainly imputable to the extension units. The treatment affected the percentage of galloylation for Nebbiolo and Barbera (*p* < 0.001). In Barbera skins (Table 1), a decrease of the G value in ozone-treated samples was found compared to their air-exposed analogous (−0.34%; *p* < 0.05 and −0.98%; *p* < 0.01, for ozone treatment at 10 and 20% WL, respectively). With regard to the dehydration level, air-exposed samples at 20% WL reached a higher percentage of galloylated constituents than Barbera fresh grapes and dehydrated at 10% WL under air exposure (+0.56% with respect to fresh grapes; *p* < 0.01). In ozone-treated samples, a decrease in the G value was observed at 10% WL and remained constant at 20% WL (−0.40 and −0.42% compared to fresh grapes, respectively; *p* < 0.01).

A different behavior of galloylated constituents in Nebbiolo skins was reported (Table 2), increasing with both the treatment and dehydration (both *p* < 0.001). This increase was more important for the ozone-treated than air-exposed samples (G, +0.40%; *p* < 0.001, for ozone treatment and +0.28%; *p* < 0.01, for air exposure, both at 20% WL, with respect to fresh grapes), and it was also consistent with the ECG content (μM). In terms of the ECG content, galloylation decreased in Barbera skins and increased in Nebbiolo during dehydration, involving a variety effect.

Chemical rearrangement in the PA structure may be responsible for subunit ratio modifications. Although an order of disappearance of skin PA extension units was evidenced (ECG > EGC > EC) by Lee,^31^ the PA modification rate is influenced by the subunit initial content and the subsequent differences in the percentage of ProDP and G may be given by other subunits disappearing faster.^31^ In contrast, the molar increase of ECG in Nebbiolo is not explained by this mechanism. PA galloylation is far to be clear in wine grapes: active genes involved in grape skins during grape ripening were recently found,^41^ but their activity during postharvest is still to be determined.

### Discriminant Analysis with Treatment and Dehydration as Factors

**Figure 3:**
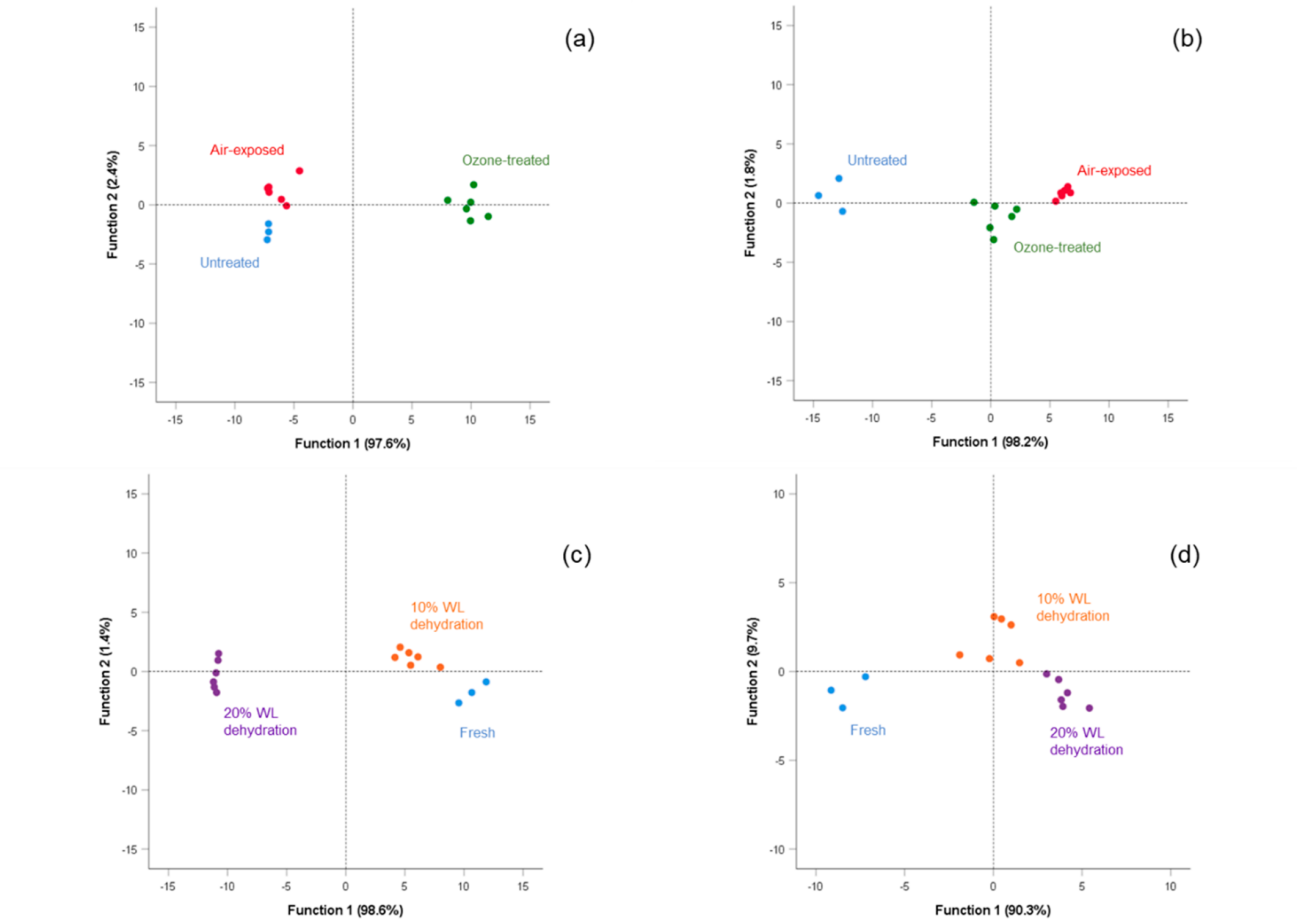
Discriminant analysis for (a and c) Barbera and (b and d) Nebbiolo wine grapes during partial dehydration up to 20% WL under air- and ozone-enriched atmosphere, attempting to separate samples replicated according to (a and b) treatment and (c and d) dehydration. WL, weight loss.

Discriminant analysis (DA; Figure 3) was performed considering the PA content and composition parameters. In all of the analyzed samples (treatments and dehydration for Barbera; panels a and c of Figure 3, respectively, and treatments and dehydration for Nebbiolo; panels b and d of Figure 3, respectively), the significant predictors among the variables reported in Tables 1 and 2 were PAs determined by phloroglucinolysis (phl-PAs), the mean degree of polymerization (mDP), the galloylation and prodelphinidin percentages (G % and ProDP %, respectively), catechin terminal and extension units (C_t_ % and C_ext_ %, respectively), and the content of epicatechin gallate (ECG, μM). These variables affected the discrimination to different extents depending upon the factors and the variety (Table 4).

**Table 4:** Standardized Coefficients of Canonical Discriminating Function^a^

	treatment effect				dehydration effect			
	Barbera		Nebbiolo		Barbera		Nebbiolo	
parameter	function 1	function 2	function 1	function 2	function 1	function 2	function 1	function 2
phl-PAs (mg/g of skin dw)	18.623	3.924	3.298	2.898	7.863	5.236	0.092	–0.852
mDP	–1.112	1.861	0.765	0.632	0.154	1.583	–0.221	0.091
G (%)	3.486	1.416	6.079	4.970	1.911	3.247	0.968	–1.727
C_t_ (%)	4.303	–0.486	0.928	–0.690	–0.373	–0.042	–0.004	1.123
ProDP (%)	–1.026	1.510	–1.843	–0.121	–3.624	1.035	–0.733	–0.174
C_ext_ (%)	0.798	–0.498	4.899	0.936	–2.430	–0.918	1.804	0.616
ECG (μM)	–12.519	–2.560	–2.794	–5.514	–1.712	–2.766	1.185	1.296

a Abbreviation: phl-PAs, total proanthocyanidins estimated by the phloroglucinolysis method; mDP, mean degree of polimerization; G, galloylation; ProDP, prodelphinidins; C_t_, catechin terminal units; C_ext_, catechin extension unit; ECG, epicatechin gallate; and dw, dry weight.

With regard to the treatment, DA allowed for the correct classification of 73.3 and 86.7% of samples in the corresponding treatment group for Barbera (Figure 3a) and Nebbiolo (Figure 3b), respectively. For Barbera, function 1a (F1a) explained 97.6% of total variance explained and was positively correlated with phl-PAs (18.623), followed by C_t_ % (4.303) and G % (3.486). In contrast, the ECG content was negatively correlated with F1a (−12.519). Ozone-treated samples were well-differentiated by F1a from the air-exposed and fresh samples. A positive correlation was evidenced for these samples with F1a, and therefore, they were characterized by a higher content of phl-PAs. Instead, the fresh and air-exposed samples, besides the lower phl-PAs, were characterized by high ECG contents. In Nebbiolo, function 1b contributed to 98.2% of the total variance explained and was described mainly by G % (6.079), C_ext_ % (4.899), and phl-PAs (3.298). Function 2b was mainly explained by the ECG content (−5.514). In Nebbiolo skins, the air-exposed samples were the most positively correlated with function 1b, showing higher G % and C_ext_ % values, followed by the ozone-treated samples and in contrast to fresh samples.

When the dehydration effect was considered, DA allowed for the correct classification of 93.3 and 80.0% of samples in the respective dehydration level for Barbera (Figure 3c) and Nebbiolo (Figure 3d), respectively. In Barbera, function 1c (F1c) accounted for the 98.6% of the total variance explained and was positively correlated with the phl-PA content (7.863) and negatively correlated with ProDP % (−3.624) and C_ext_ % (−2.430). Samples dehydrated at 20% WL were positioned in the negative part of F1c and, therefore, were characterized by lower phl-PAs and a higher contribution of ProDP % in the compositional traits. In contrast, fresh samples and those dehydrated at 10% WL reported more similar features, even if they can be differentiated according to function 2c by galloylation (G %, 3.247; ECG content, −2.766). In Nebbiolo, functions 1d and 2d accounted for 90.3 and 9.7% of total variance explained, respectively. The first function well discriminated the samples dehydrated at different levels from the fresh grapes, and WL levels were positively correlated with C_ext_ % and ECG content (1.804 and 1.185, respectively). Between the two WL levels, function 2d discriminated according to compositional variables: samples dehydrated at 10% WL were in the positive part of the graph reporting higher C_t_ % (1.123), whereas those dehydrated at 20% WL were correlated with a higher G % value (−1.727).

To conclude, both partial grape dehydration and long-term ozone treatment modified to different extents the PA content and composition depending strongly upon the variety. Nevertheless, these two factors led to significant differences in the two varieties studied and contributed efficiently in sample discrimination for each variety. In general, a decrease of the total PA content during the dehydration was found for Barbera, while the continuous ozone treatment limited PA loss. In fact, a significant interaction was found among dehydration and ozone exposure effects on Barbera wine grapes. Other important modifications were given by the compositional traits. In this variety, the combined effect of dehydration and ozone treatment has an additional advantage, allowing for the increase of the percentage of prodelphinidins (ProDP %) and decrease of galloylation (G %). These two modifications are of great relevance for the presence of smoother and less astringent skin tannins, respectively. Also, mDP is recognized as predictor of astringency, and low values are associated with astringency softening. In our findings, Barbera skin mDP increased during dehydration but ozone-treated samples showed significantly lower values than those exposed to air.

For Nebbiolo skins, the most relevant differences were reported in the PA composition, although most compositional parameters were barely affected. Contrary to what was observed for Barbera, galloylation increased during dehydration, particularly when it was performed under ozone exposure. This may be relevant in winemaking practices. Nebbiolo skins are much richer in PAs than Barbera, and although the total PA content was not influenced by dehydration or ozone treatment and skin galloylation is generally low, the higher presence of galloylated compounds is strongly correlated with wine astringency.

Generally, PA synthesis and evolution are far to be fully understood in grapes. No induction of fundamental genes involved in the PA synthesis was reported during postharvest dehydration, whereas ozone-induced PA modifications were still not investigated. Nevertheless, an increased expression of phenylpropanoid genes, induced by oxidative stress, was previously observed,^42^ as occurs during dehydration. PA chemical modifications should be considered in terms of hydrolysis as well as of inter- and intramolecular oxidative coupling. They particularly affect the content of monomeric constituents, degree of polymerization, and structure. Therefore, further studies may be conducted to better understand these oxidative mechanisms in grapes during postharvest processes. The knowledge of the PA profile and content in the variety tested as well as PA modifications during grape postharvest treatments may allow for the better exploitation of different winemaking strategies influencing the final wine quality and market value.
